# Supplementary material for: Single-nucleus RNA-seq identifies transcriptional heterogeneity in multinucleated skeletal myofibers
Source: Nat Commun. 2020 Dec 11;11:6374. doi: 10.1038/s41467-020-20063-w (PMC7733460; doi:10.1038/s41467-020-20063-w)
Supplement: Supplementary file 1 — Supplementary Information [file 41467_2020_20063_MOESM1_ESM.pdf]

Supplementary Information

Single-nucleus RNA-seq identifies transcriptional heterogeneity in multinucleated skeletal myofibers

Petrany MJ, et al.

This PDF file includes:

Supplementary Figure 1
Supplementary Figure 2
Supplementary Figure 3
Supplementary Figure 4
Supplementary Figure 5
Supplementary Figure 6
Supplementary Figure 7
Supplementary Figure 8
Supplementary Figure 9
Supplementary Figure 10
Supplementary Figure 11
Supplementary Figure 12
Supplementary Figure 13
Supplementary Figure 14
Supplementary Figure 15
Supplementary Figure 16
Supplementary Table 1

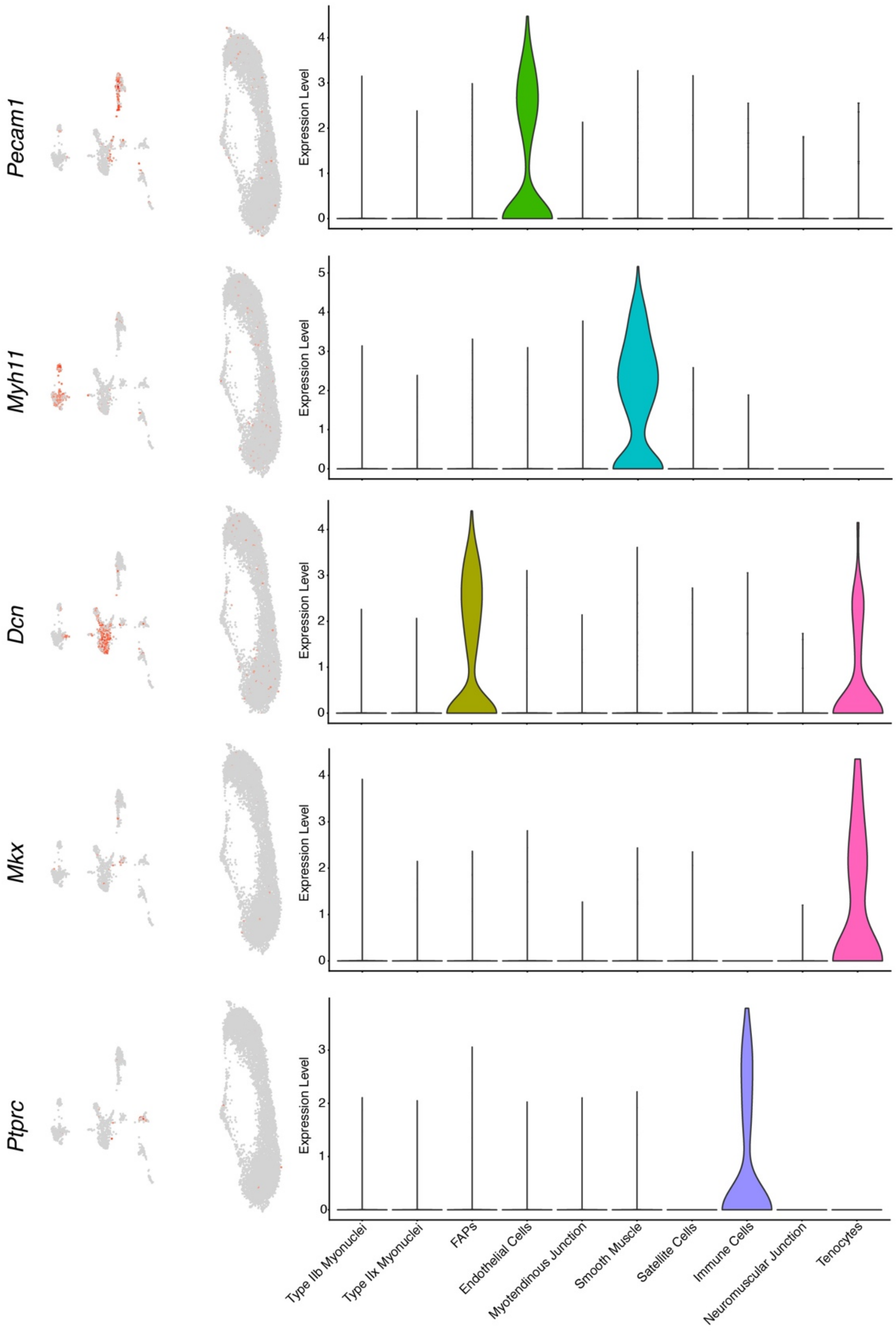

Supplementary Fig. 1. Identification of non-muscle nuclei in 5-month tibialis anterior snRNA-seq dataset. Feature and violin plots for markers of endothelial cells (*Pecam1*), smooth muscle (*Myh11*), fibroadipogenic progenitors (FAPs) (*Dcn*), tenocytes (*Mkx*), and immune cells (*Ptprc*). Type IIb and Type IIx myonuclei were combined with their intermediate clusters for generation of violin plots.

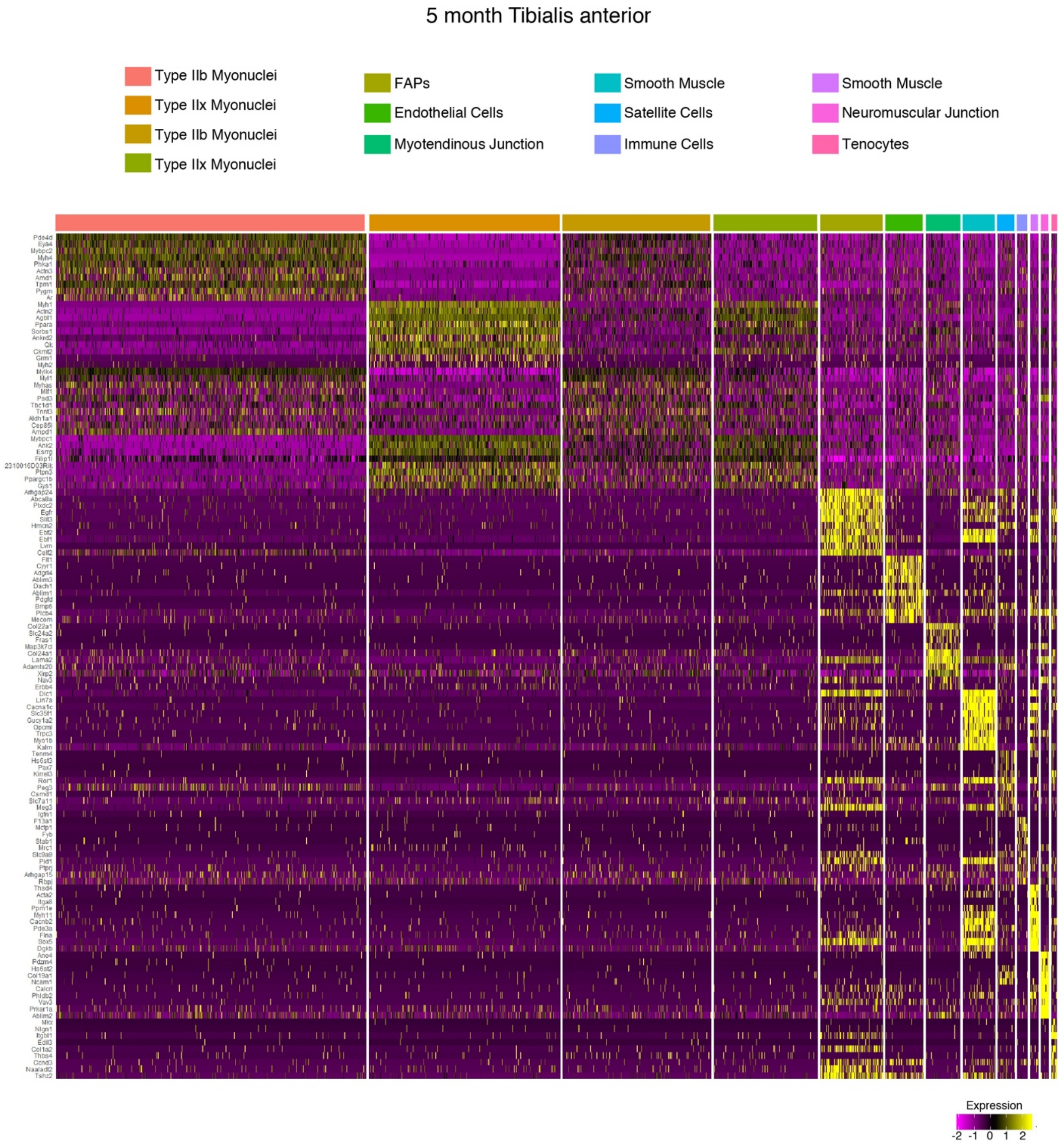

Supplementary Fig. 2. Top differentially expressed marker genes for clusters in 5-month tibialis anterior muscle. Normalized expression (Z-score) heatmap showing the top differentially expressed genes among the 13 clusters identified by snRNA-seq. Columns are individual nuclei belonging to each of the clusters labeled above.

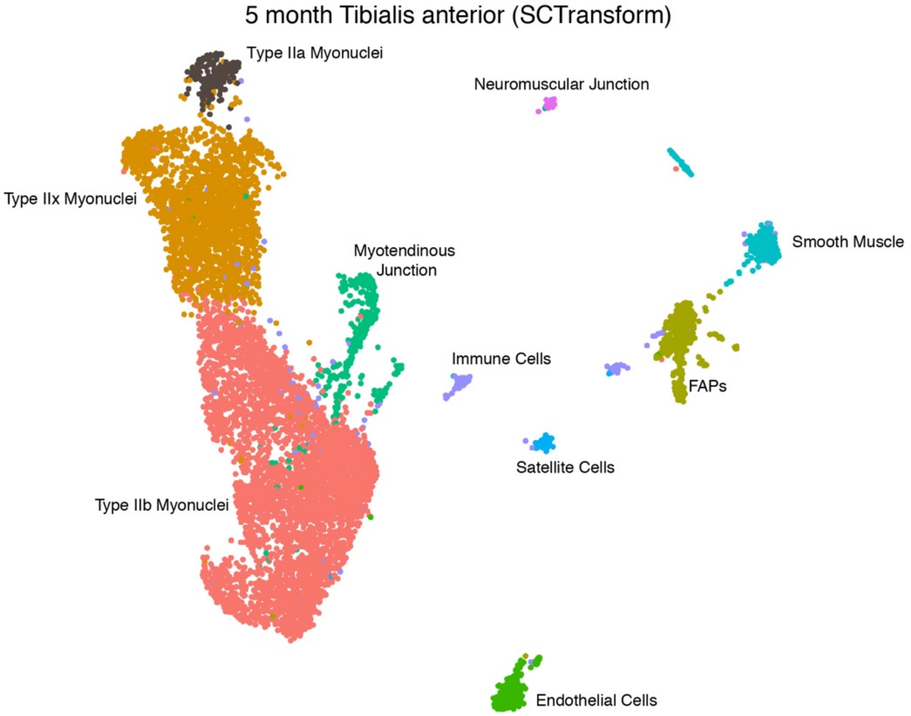

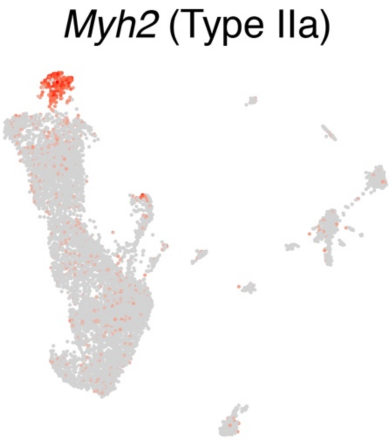

Supplementary Fig. 3. Higher dimensionality clustering of nuclear sequencing data through use of the SCTransform function. This analysis revealed the presence of myonuclei positive for *Myh2* (Type IIa). Type IIa fibers are known to comprise a small percentage of the fiber types in the tibialis anterior.

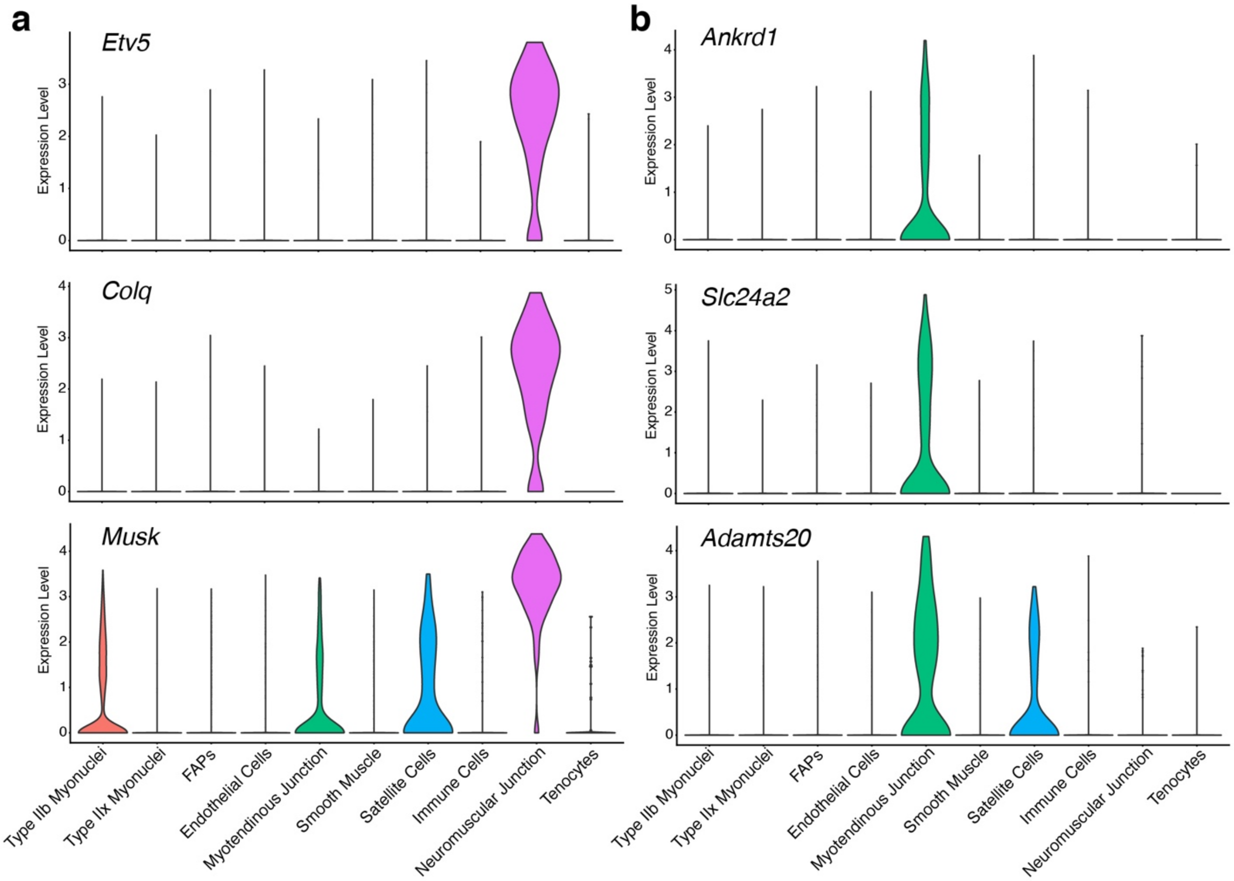

Supplementary Fig. 4. Identification of known genes and genes previously not associated with the neuromuscular junction (NMJ) and myotendinous junction (MTJ). a, Violin plots for the NMJ-enriched genes including *Etv5*, *Colq*, and *Musk*. **b**, Violin plots for *Ankrd1*, *Slc24a2*, and *Adamts20*, which we identified as being enriched in MTJ myonuclei.

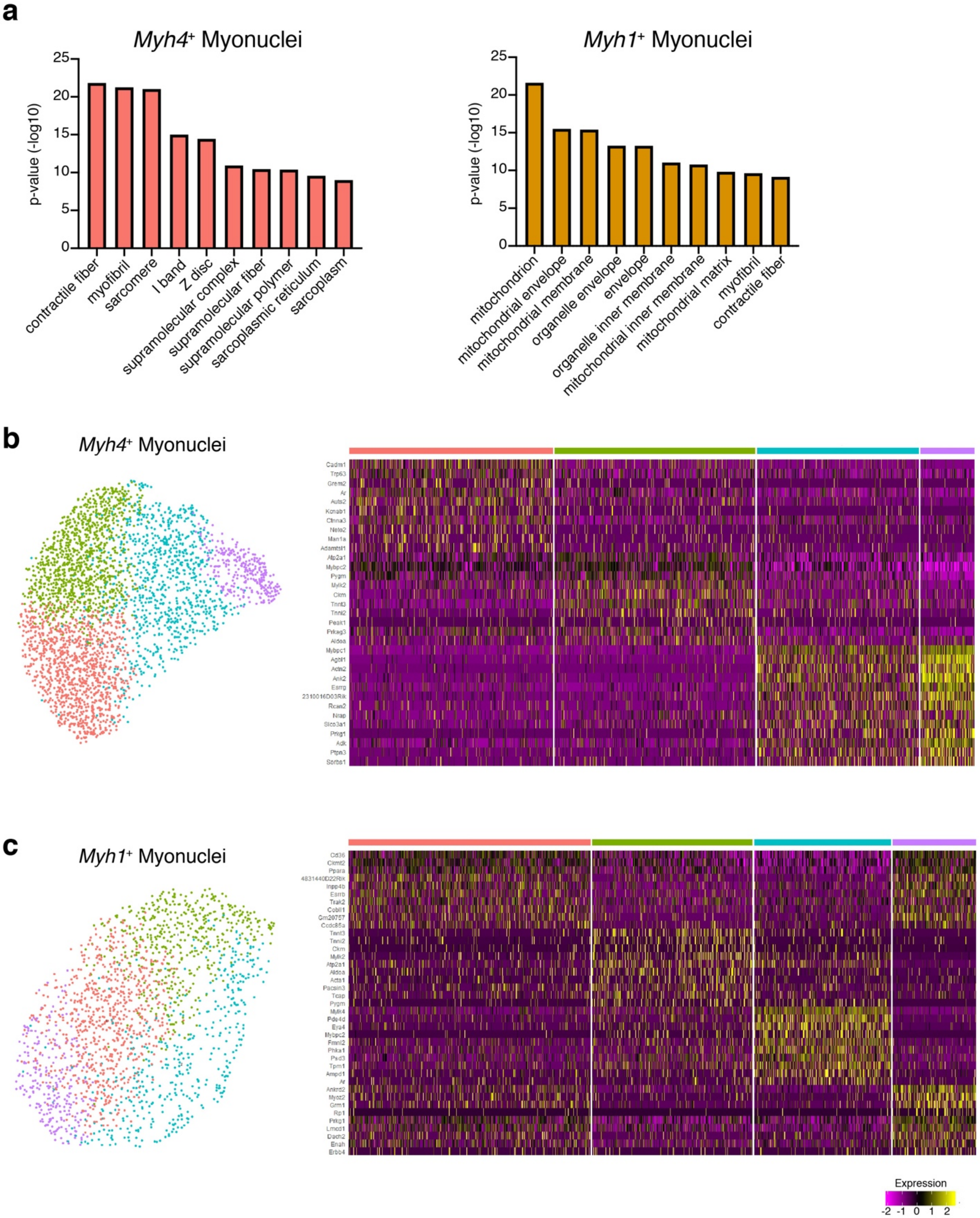

Supplementary Fig. 5. Fiber type-specific myonuclei show diversity of transcriptional states. a, Gene ontology analysis of *Myh4*$^{+}$/*Myh1*$^{-}$/*Myh2*$^{-}$ (exclusively Type IIb) myonuclei (left panel) and *Myh1*$^{+}$/*Myh4*$^{-}$/*Myh2*$^{-}$ (exclusively Type IIx) myonuclei (right panel) **b**, Sub-clustering with UMAP projection and normalized expression (Z-score)

heatmap generation of *Myh4*⁺ myonuclei. **c**, UMAP and heatmap of *Myh1*⁺ myonuclei. Both myonuclear types show sub-clusters of divergent gene expression states. Columns are individual nuclei.

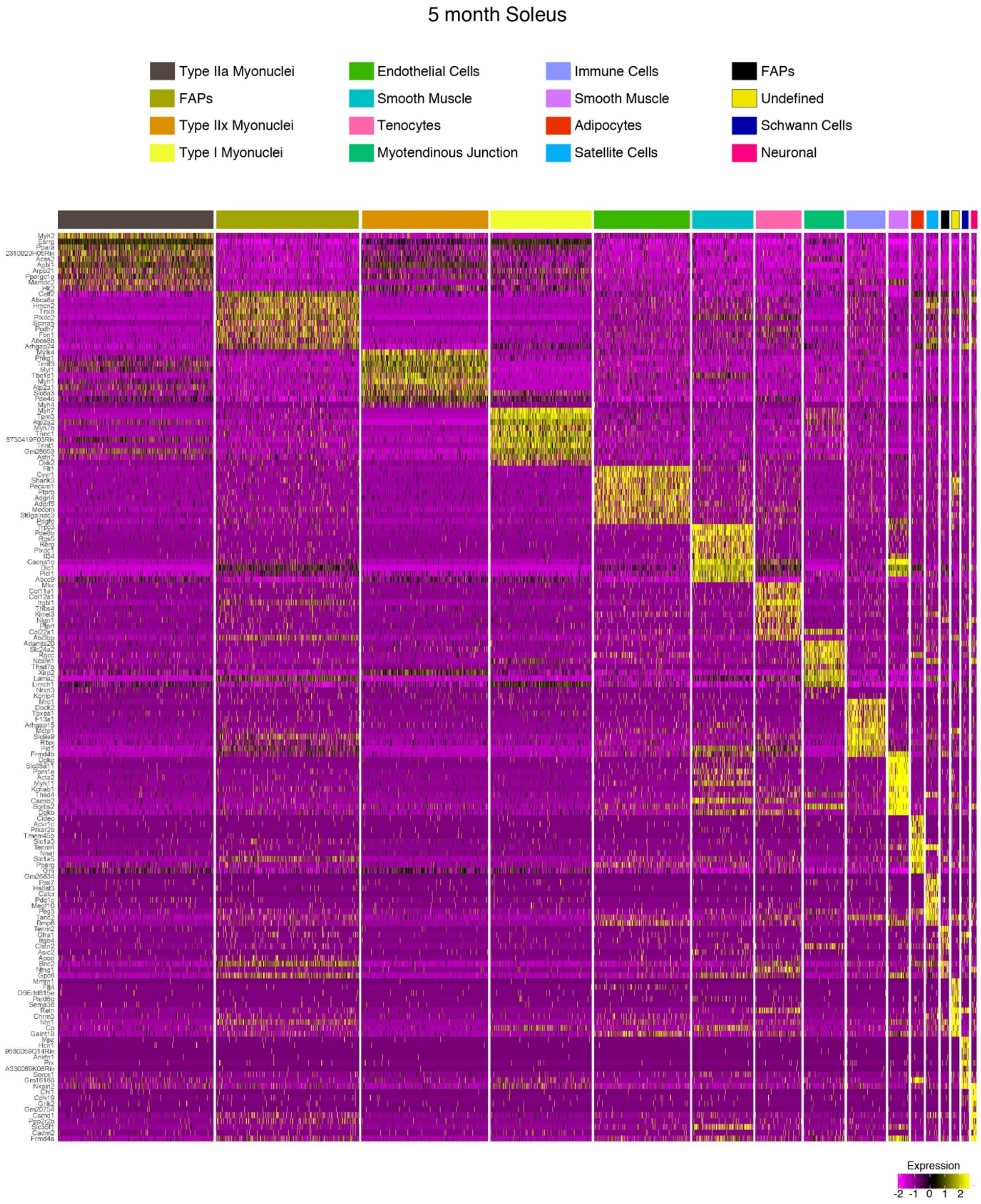

Supplementary Fig. 6. Top differentially expressed marker genes for clusters in 5-month soleus muscle. Normalized expression (Z-score) heatmap showing the top differentially expressed genes among the 16 clusters identified by snRNA-seq. Columns are individual nuclei belonging to each of the clusters labeled above.

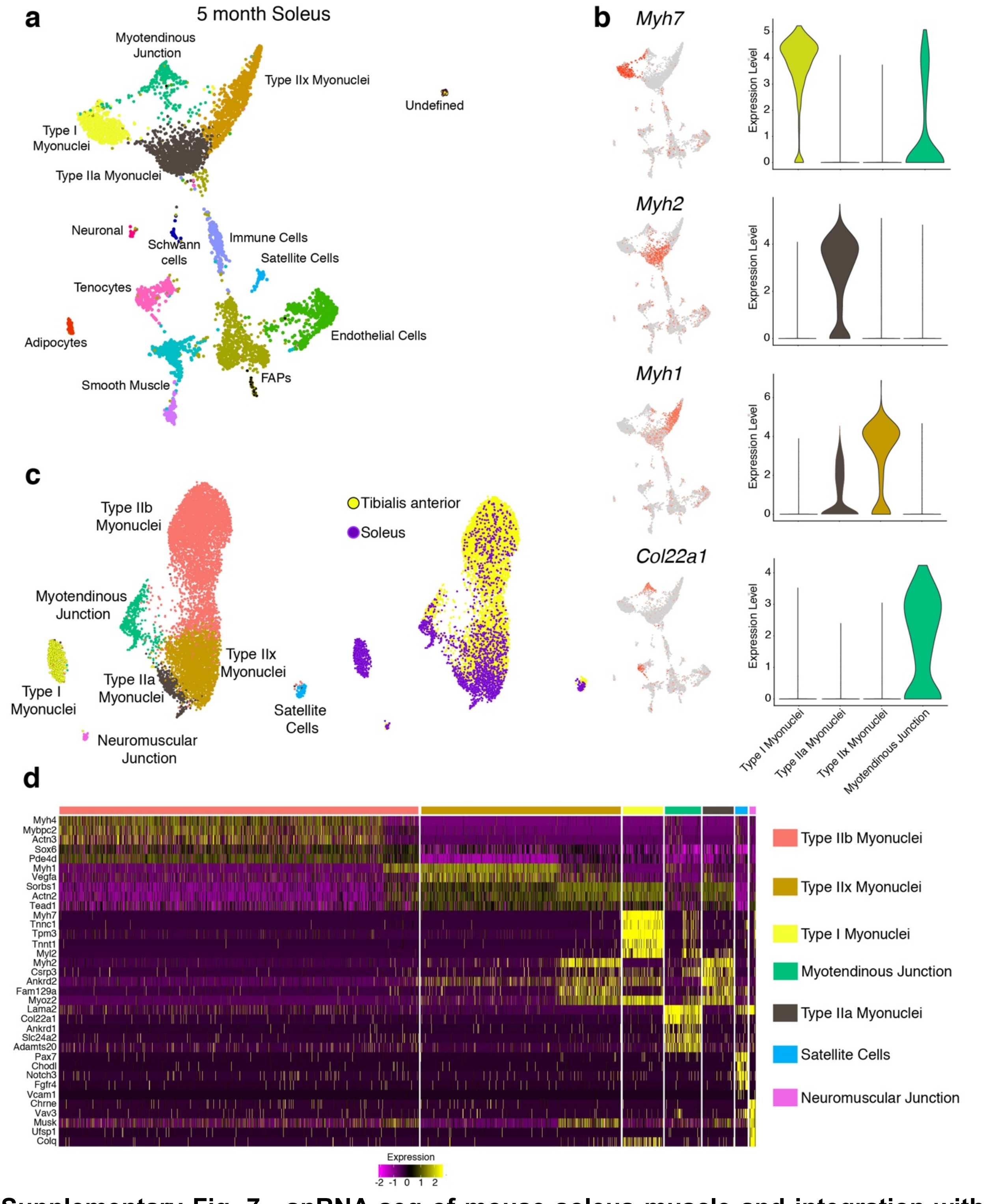

Supplementary Fig. 7. snRNA-seq of mouse soleus muscle and integration with tibialis anterior reveal transcriptomes of all fiber types. **a**, Unbiased clustering of nuclei represented in a UMAP from the soleus muscle showing Type I, Type IIa, and Type IIx myonuclei. **b**, Feature and violin plots for *Myh7*, *Myh2*, and *Myh1* showing enriched expression in the expected fiber types. *Col22a1* also marks the myotendinous junction in

slow-twitch muscle. **c**, Integration of myonuclear populations from the tibialis anterior and soleus muscles showing that Type I myonuclei are the most divergent myonuclear population. The right panel shows if the nuclei in the left panel originates from the tibialis anterior or soleus. **d**, Heatmap showing the top genes expressed in the muscle-related nuclear populations present in the integrated tibialis anterior/soleus dataset.

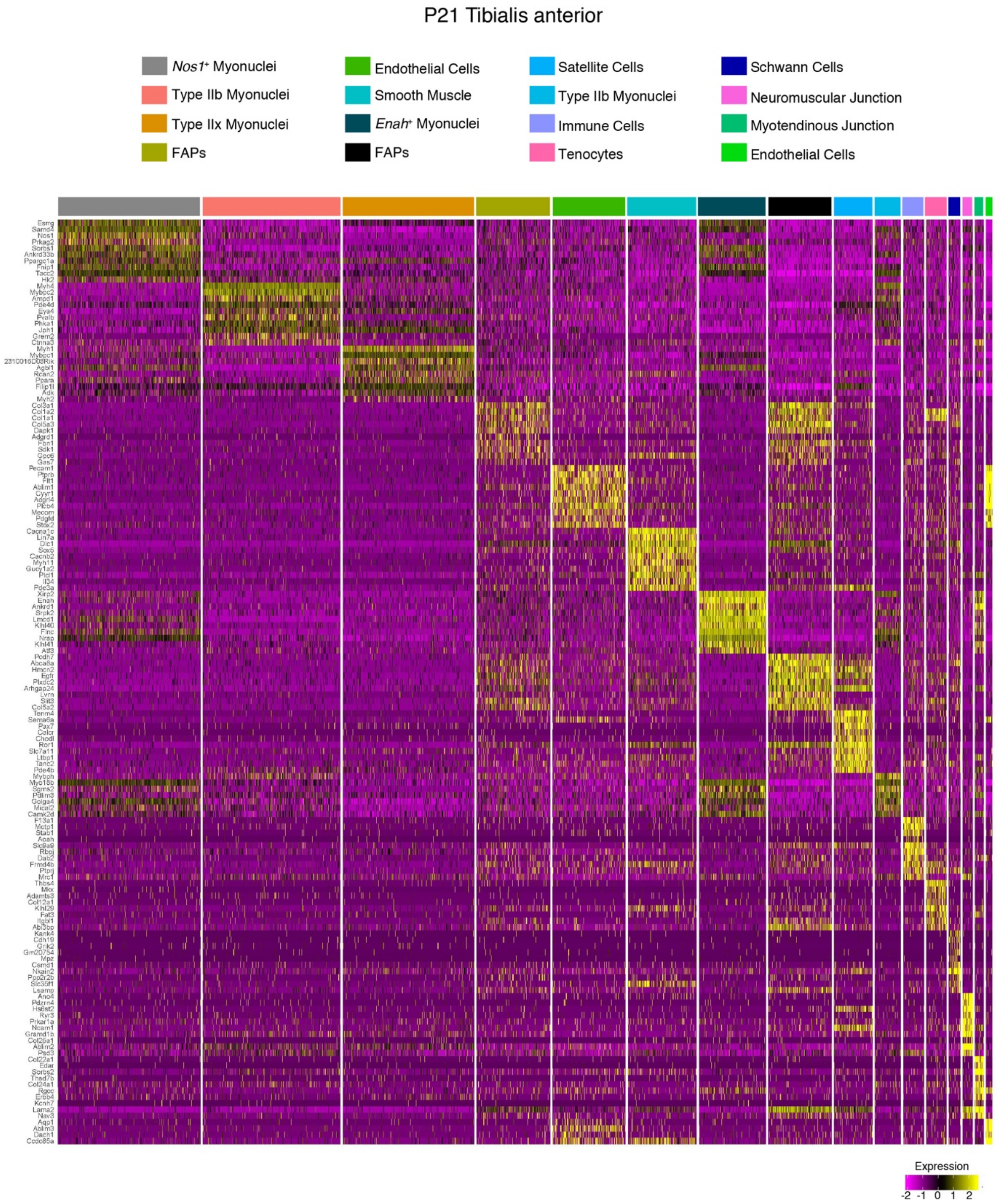

Supplementary Fig. 8. Top differentially expressed marker genes for clusters in postnatal day 21 tibialis anterior muscle. Normalized expression (Z-score) heatmap showing the top differentially expressed genes among the 16 clusters identified by snRNA-seq. Columns are individual nuclei belonging to each of the clusters labeled above.

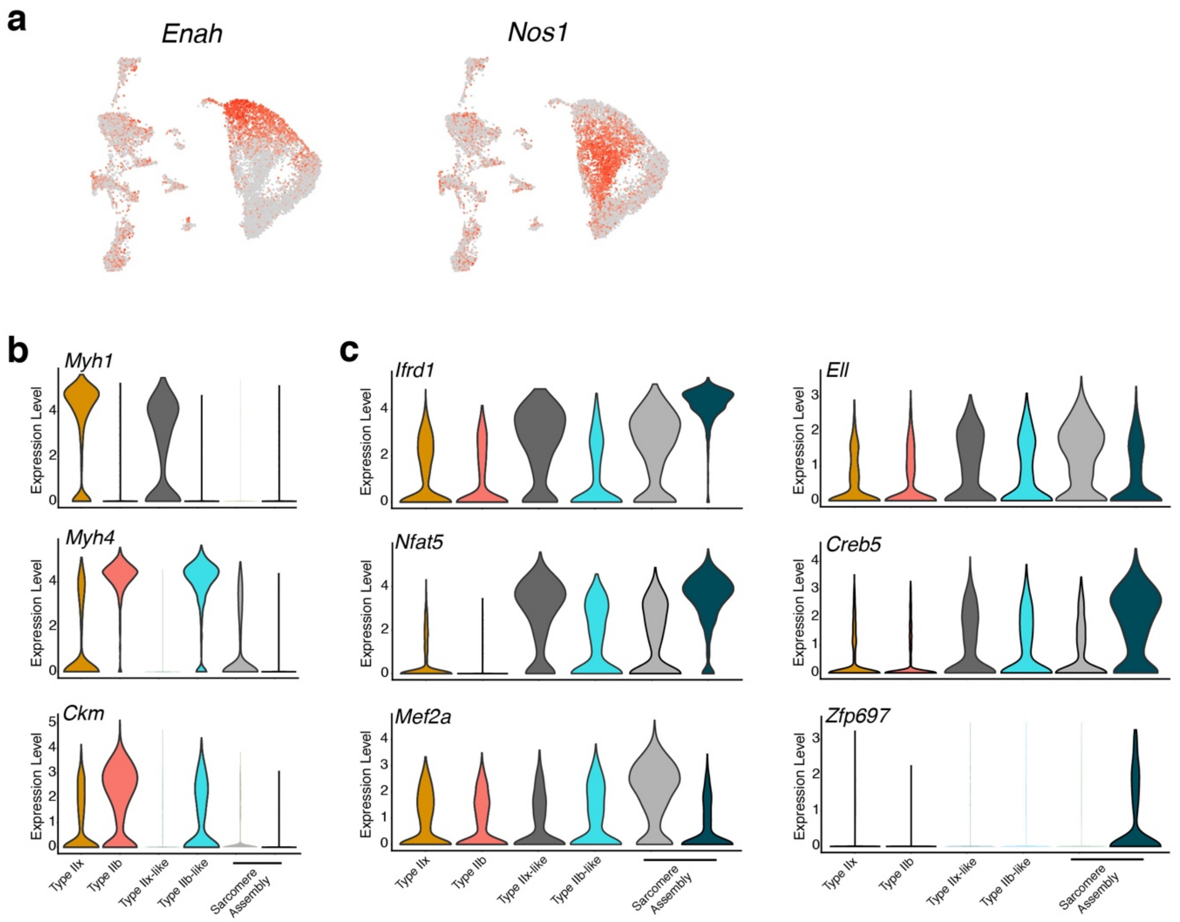

Supplementary Fig. 9. Sarcomere assembly myonuclear states in P21 muscle display non-uniform gene expression. **a**, Feature plots for *Enah* and *Nos1* from the P21 sample. **b**, Violin plots for *Myh1*, *Myh4*, and *Ckm* show low or minimal expression of these genes in the sarcomere assembly myonuclear populations. **c**, Differential expression of transcription factors in sarcomere assembly myonuclei compared to myonuclei that highly express myosins.

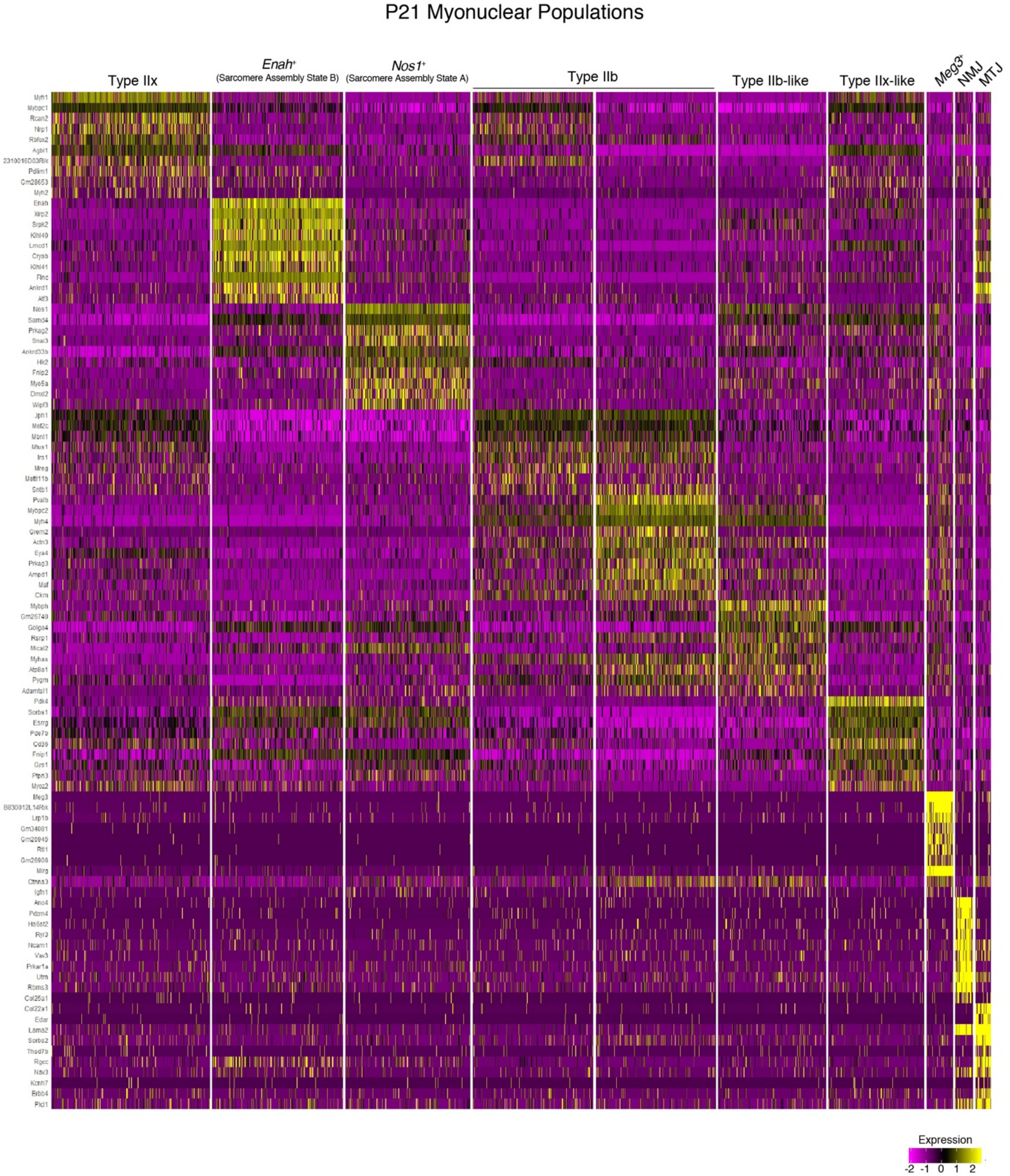

Supplementary Fig. 10. Heterogeneous pattern of skeletal muscle-specific gene signatures in P21 tibialis anterior muscle. Heatmap showing distinct populations of myonuclei in developing muscle, including *Enah*+ and *Nos1*+ clusters with negligible expression of myosin heavy chains (*Myh4* and *Myh1*) but a distinct enrichment of muscle-specific factors involved in sarcomere formation and myofibrillogenesis.

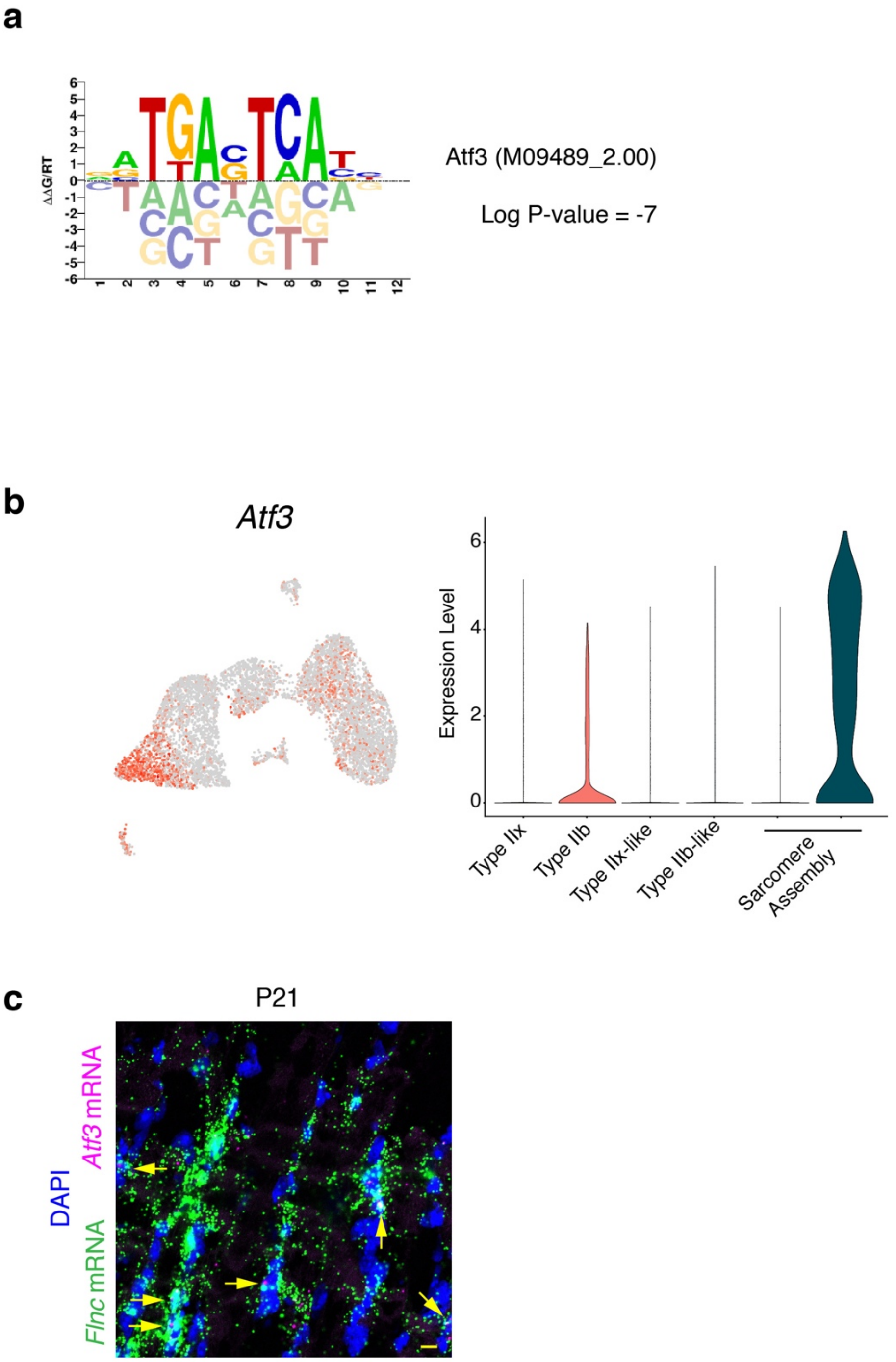

Supplementary Fig. 11. Atf3 as a putative transcriptional regulator of the sarcomere assembly *Enah*⁺ myonuclear program. a, Promoter sites of *Enah*⁺ myonuclear marker genes are enriched for Atf3-binding motifs (HOMER motif detection analysis). 30% of genes in the *Enah*⁺ population contain predicted Atf3 binding sites in their promoter region (-1000 – 1000). **b**, Feature plot and violin plot showing that *Atf3* is upregulated at the transcriptional level in *Enah*⁺ sarcomere assembly state B. **c**, smRNA-FISH for *Flnc* and *Atf3* shows localization of *Atf3* transcripts in nuclei with high levels of *Flnc* transcription (n=4). Scale bar: 10 μm.

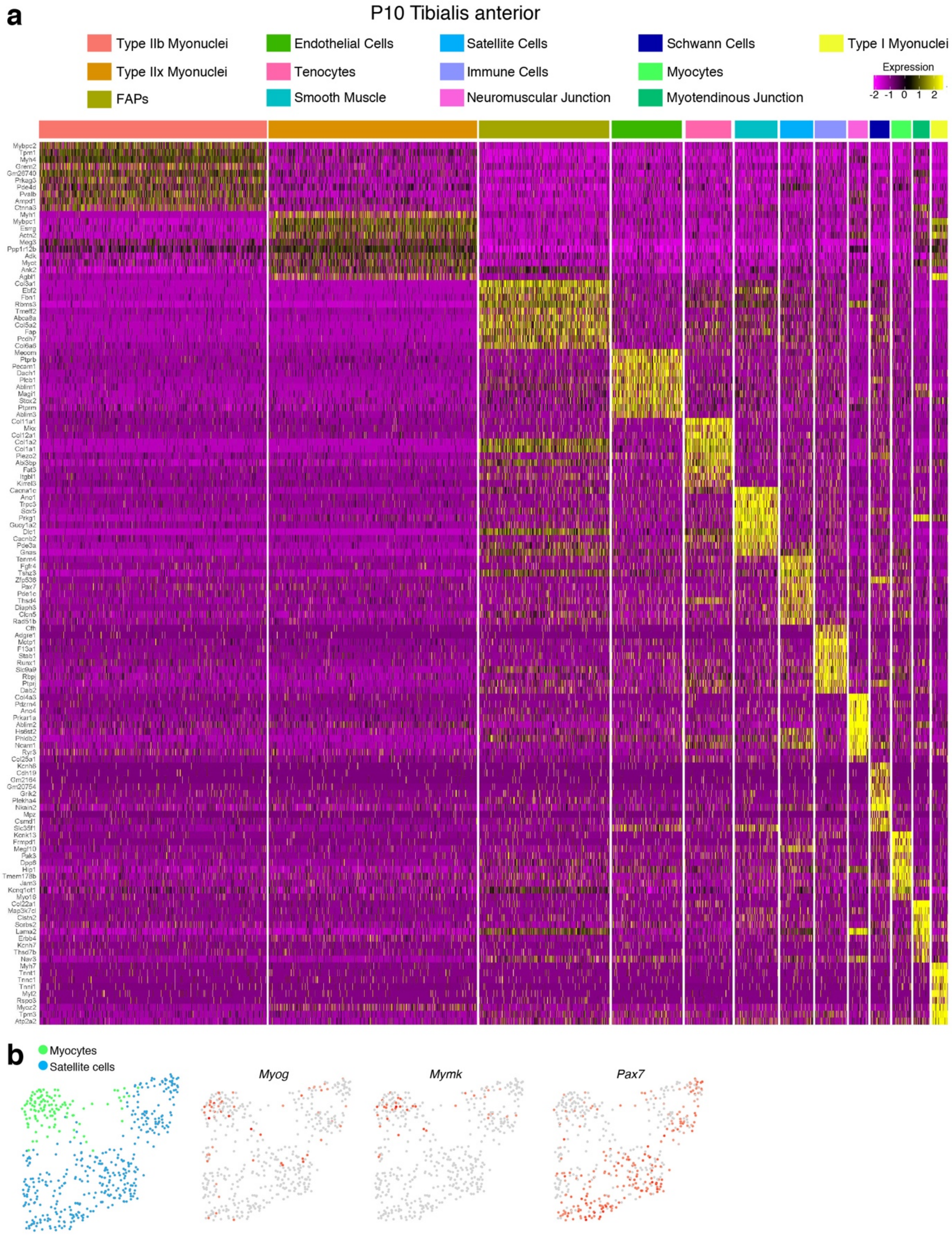

Supplementary Fig. 12. Top marker genes for clusters in postnatal day 10 tibialis anterior muscle and presence of a fusogenic myocyte population. a, Normalized expression (Z-score) heatmap of P10 tibialis anterior showing the top differentially expressed genes among the 13 clusters identified by snRNA-seq. Columns are individual

nuclei belonging to each of the clusters labeled above. **b**, UMAP representation (left panel) after sub-clustering satellite cells and myocytes from the P10 dataset. Panels on right are feature plots for *Myog*, *Mymk*, and *Pax7*, which confirmed the myocyte population.

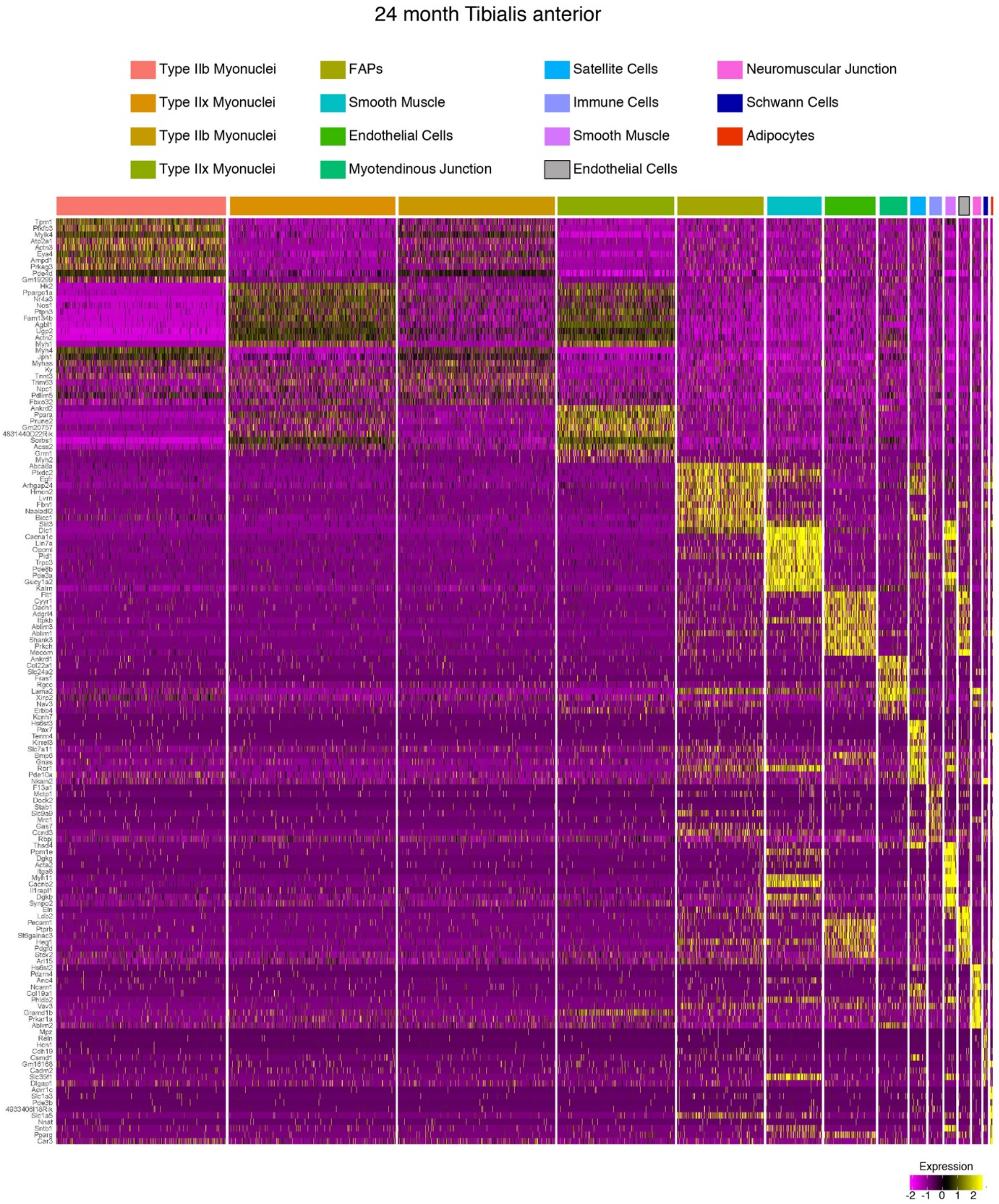

Supplementary Fig. 13. Top differentially expressed marker genes for clusters in 24-month tibialis anterior muscle. Normalized expression (Z-score) heatmap showing the top differentially expressed genes among the 15 clusters identified by snRNA-seq. Columns are individual nuclei belonging to each of the clusters labeled above.

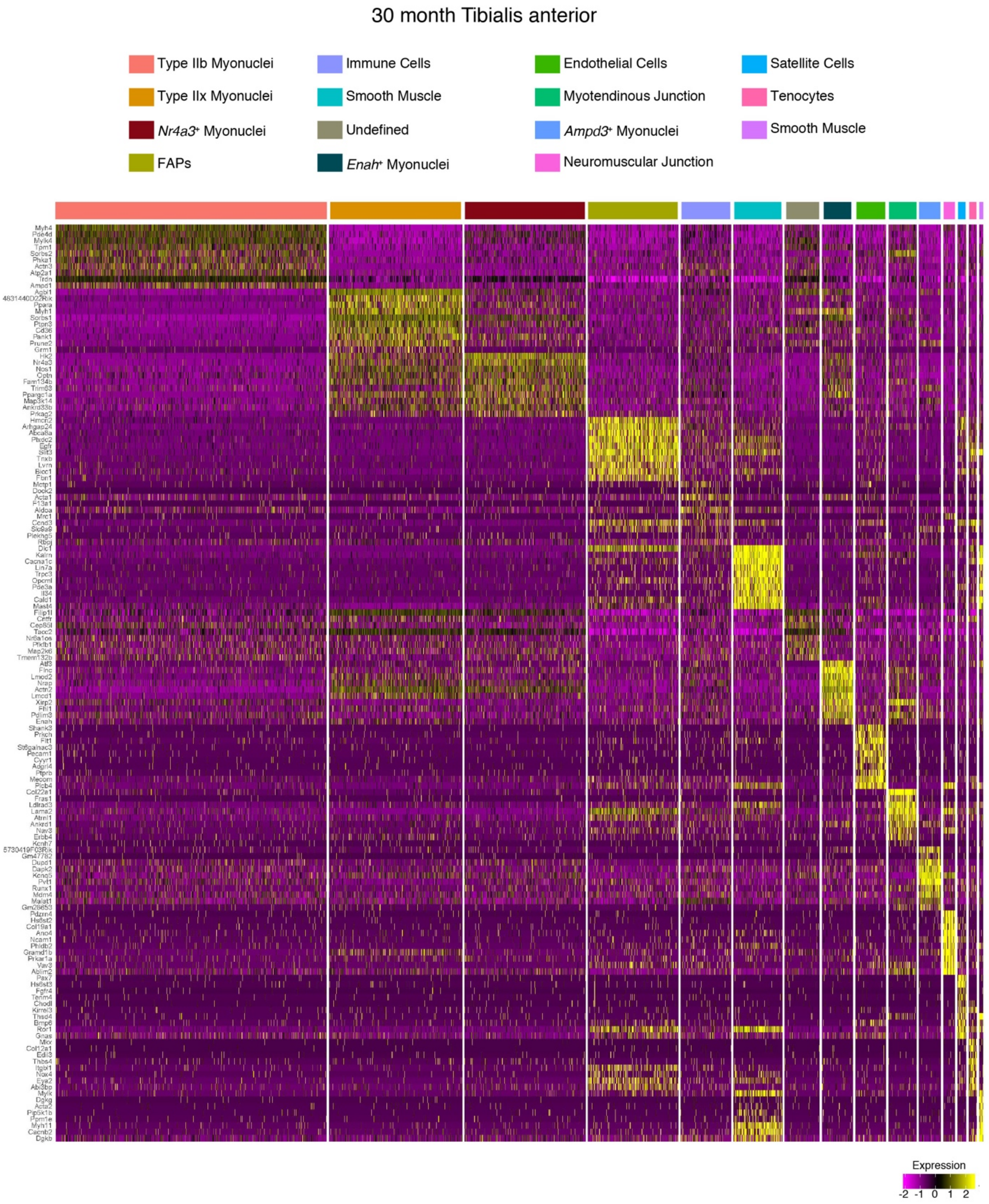

Supplementary Fig. 14. Top differentially expressed marker genes for clusters in 30-month tibialis anterior muscle. Normalized expression (Z-score) heatmap showing the top differentially expressed genes among the 15 clusters identified by snRNA-seq. Columns are individual nuclei belonging to each of the clusters labeled above.

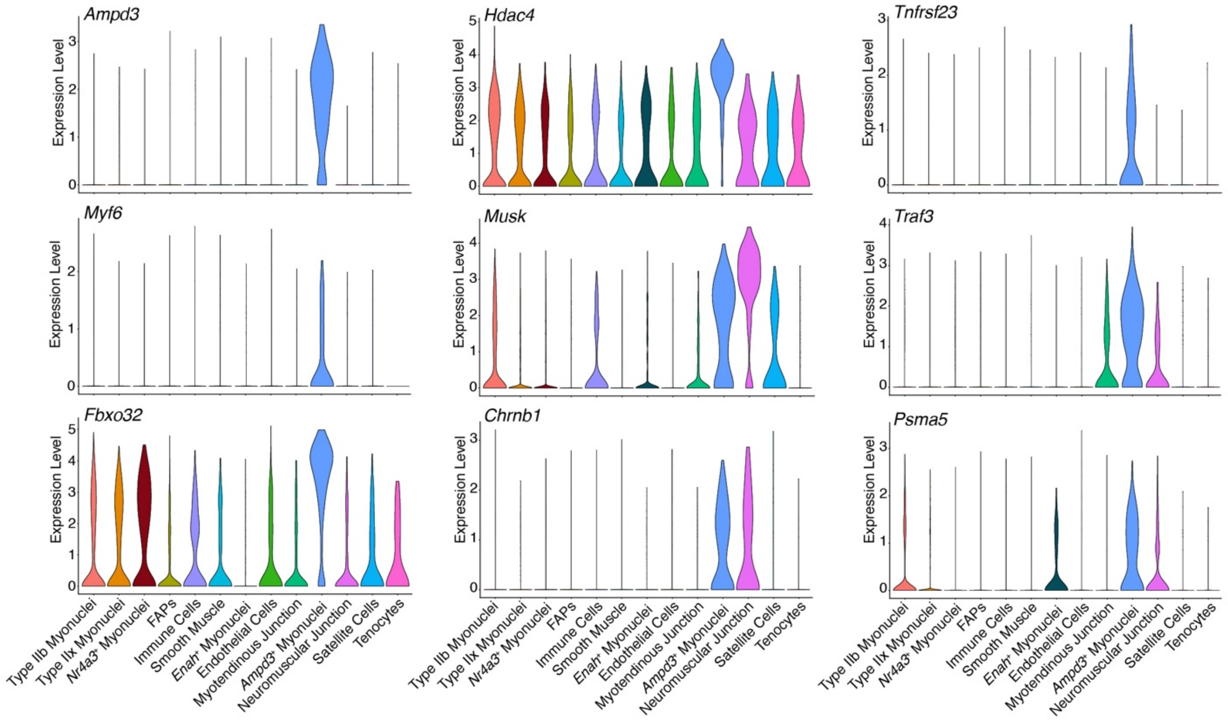

Supplementary Fig. 15. Disruption of gene expression in aged myonuclei. a, Violin plots from the nuclei isolated from 30-month old muscle showing *Ampd3*$^{+}$ myonuclei are expressing muscle and NMJ genes, but also are enriched for genes associated with inflammation, cell death, and the proteasome.

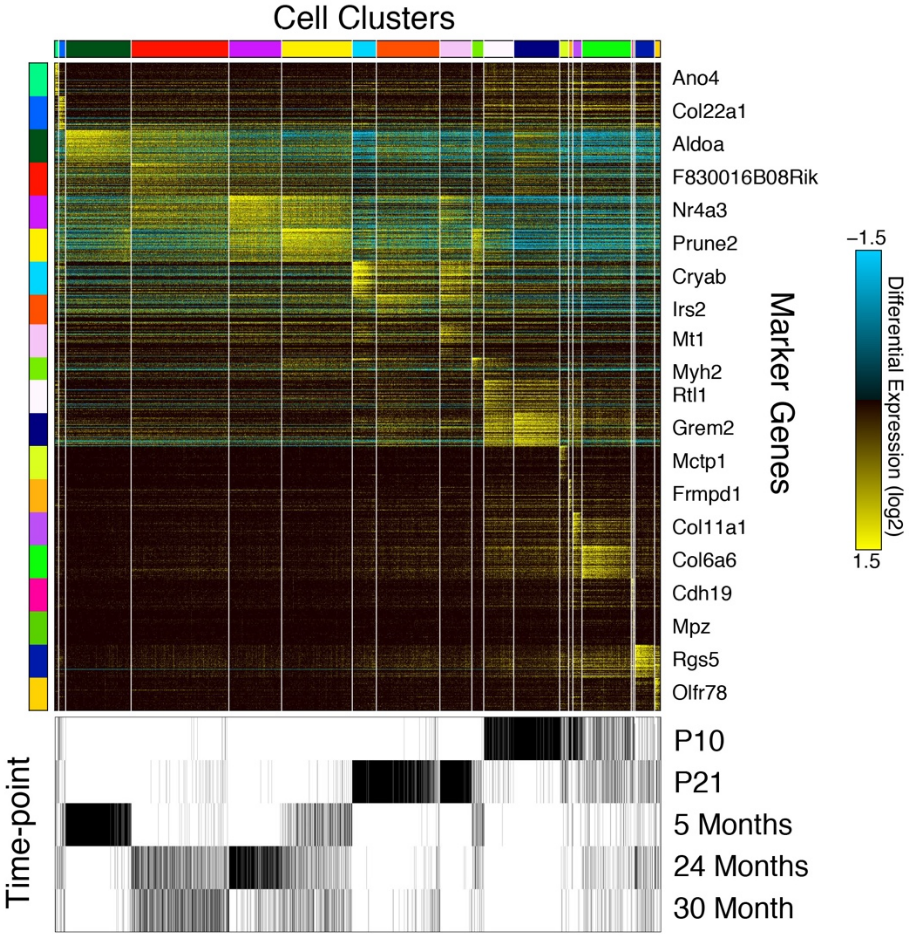

Supplementary Fig. 16. Myonuclei from each time-point intermix to varying degrees based on temporal proximity. Heatmap of gene expression clusters (n=20) identified using the unsupervised ICGS2 workflow of all snRNA-Seq captures. The top 60 marker genes are displayed for each corresponding cell cluster. The time-points associated with each cell are indicated by tickmarks below the heatmap. Enriched gene-sets from diverse single-cell and bulk reference profiles.

Supplementary Table 1. Primer sequences used for quantitative real-time PCR.

GAPDH F	TGCGACTTCAACAGCAACTC
GAPDH F	GCCTCTCTTGCTCAGTGTCC
Musk F	TGAAGCTGGAAGTGGAGGTTTT
Musk R	GCAGTAGGGTTACAAAGGAA
Vav3 F	GGGTAATAGAACAGGCACAGC
Vav3 R	GCCATTTACTTCACCTCTCCAC
Gramd1b F	ATCAGCTGTGTTCTGGTGCT
Gramd1b R	GGGTAACCTTTCCTGGAGCC
Ufsp1 F	GAAAACACCAGGAACCCGGA
Ufsp1 R	AGGTAGTGGCCTGAGAGGAG
B4galnt3 F	ACCCCCTGAACCACAGGTAT
B4galnt3 R	GGAGATTCGGATCAACGGCT
Nestin F	TGTCCCTTAGTCTGGAAGTGG
Nestin R	GGTGTCTGCAAGCGAGAGTT
AChR E F	TTCGCTCCCAGACCTACAATG
AChR E R	TCGTCATCCACGGCAAAGA
Ache F	CTCCCTGGTATCCCCTGCATA
Ache R	GGATGCCCAGAAAAGCTGAGA
Pdzrn4 F	CGGCCAACATACGGCATGA
Pdzrn4 R	GGCCATGATGTGTTCAAAAGTG
Lrfn5 F	TGTTTCTCATTGGCATAGCTGT
Lrfn5 R	TGGTGGAACAAATAGAAGCCCT
D430041D05Rik F	ACCCTCCACTCACCATTGC
D430041D05Rik R	TGTAGCTTCCCAAGGTGGTAG
Ckm F	GCCATGTGATTGTTGTGCTT
Ckm R	ACAGACACTCAGGAGCCAGC
Myh4 F	GCAGGACTTGGTGGACAAAC
Myh4 R	ACTTGGCCAGGTTGACATTG
Tnnt3 F	GGAACGCCAGAACAGATTGG
Tnnt3 R	TGGAGGACAGAGCCTTTTTCTT
