# Supplementary material for: Single-nucleus RNA-seq identifies transcriptional heterogeneity in multinucleated skeletal myofibers
Source: Nat Commun. 2020 Dec 11;11:6374. doi: 10.1038/s41467-020-20063-w (PMC7733460; doi:10.1038/s41467-020-20063-w)
Supplement: Supplementary file 6 — Reporting Summary [file 41467_2020_20063_MOESM6_ESM.pdf]

nature research

Corresponding author(s): Douglas Millay
Last updated by author(s): 10/12/2020

Reporting Summary

Nature Research wishes to improve the reproducibility of the work that we publish. This form provides structure for consistency and transparency in reporting. For further information on Nature Research policies, see Authors & Referees and the Editorial Policy Checklist .

Statistics

For all statistical analyses, confirm that the following items are present in the figure legend, table legend, main text, or Methods section.

n/a	Confirmed	
☐	☒	The exact sample size (*n*) for each experimental group/condition, given as a discrete number and unit of measurement
☐	☒	A statement on whether measurements were taken from distinct samples or whether the same sample was measured repeatedly
☐	☒	The statistical test(s) used AND whether they are one- or two-sided<br>*Only common tests should be described solely by name; describe more complex techniques in the Methods section.*
☒	☐	A description of all covariates tested
☒	☐	A description of any assumptions or corrections, such as tests of normality and adjustment for multiple comparisons
☐	☒	A full description of the statistical parameters including central tendency (e.g. means) or other basic estimates (e.g. regression coefficient) AND variation (e.g. standard deviation) or associated estimates of uncertainty (e.g. confidence intervals)
☐	☒	For null hypothesis testing, the test statistic (e.g. *F*, *t*, *r*) with confidence intervals, effect sizes, degrees of freedom and *P* value noted<br>*Give P values as exact values whenever suitable.*
☒	☐	For Bayesian analysis, information on the choice of priors and Markov chain Monte Carlo settings
☐	☒	For hierarchical and complex designs, identification of the appropriate level for tests and full reporting of outcomes
☒	☐	Estimates of effect sizes (e.g. Cohen's *d*, Pearson's *r*), indicating how they were calculated

Our web collection on statistics for biologists contains articles on many of the points above.

Software and code

Policy information about availability of computer code

Data collection	No software was used in the collection of primary data. For software packages used in analysis of raw sequencing data and statistics, see below or Methods section.
Data analysis	Raw sequencing data of all samples were processed using the cellRanger workflow (version 3.1.0). We corrected for ambient background RNA by filtering with the R package SoupX (version 0.3.0). Further data analysis was carried out using the R (version 3.6.1) package Seurat (version 3.1.0). To assess integration of the data, we used ICGS2 (http://altanalyze.org). For transcription factor binding motif analysis, we used the HOMER motif enrichment algorithm (v4.11). Additional details are available in our Methods section. Code is available from https://github.com/MillayLab/single-myonucleus.<br><br>Other software used for data anlysis included:<br>GraphPad Prism 8<br>Microsoft Excel 16.4<br>FIJI 2.0.0-rc-64/1.52e

For manuscripts utilizing custom algorithms or software that are central to the research but not yet described in published literature, software must be made available to editors/reviewers. We strongly encourage code deposition in a community repository (e.g. GitHub). See the Nature Research guidelines for submitting code & software for further information.

Data

Policy information about availability of data

All manuscripts must include a data availability statement. This statement should provide the following information, where applicable:

- Accession codes, unique identifiers, or web links for publicly available datasets
- A list of figures that have associated raw data
- A description of any restrictions on data availability

Data availability. Raw sequencing data are deposited in GEO (GSE147127) [https://www.ncbi.nlm.nih.gov/geo/query/acc.cgi?acc=GSE147127]. Additional data files and metrics have been deposited at https://www.synapse.org/#!Synapse:syn21676145. Source data are provided with this paper.
For transcription factor motif analysis, we used the CisBP database (build 2.0).

Field-specific reporting

Please select the one below that is the best fit for your research. If you are not sure, read the appropriate sections before making your selection.

☒ Life sciences ☐ Behavioural & social sciences ☐ Ecological, evolutionary & environmental sciences

For a reference copy of the document with all sections, see nature.com/documents/nr-reporting-summary-flat.pdf

Life sciences study design

All studies must disclose on these points even when the disclosure is negative.

Sample size	No sample size calculation was performed prior to experiments. We initially chose sample size based on previous experience and the sample size is sufficient to achieve statistical significance at the reported effect sizes. For snRNA-seq experiments, at least 5000 nuclei were sequenced for each dataset, based on standard 10X Chromium parameters, typical capture efficiency, and desired sequencing depth.
Data exclusions	No data were excluded from our analyses.
Replication	For smFISH and cell culture/siRNA knockdown experiments, as well as mouse denervation experiments, reported data is from multiple independent replications with control groups within each experiment. All replications for all experiments were successful.
Randomization	For AChR cluster quantification experiments and smFISH quantification experiments, random fields were imaged for all groups. Mice were randomly placed into experimental groups.
Blinding	All image analysis was performed in a blinded fashion.

Reporting for specific materials, systems and methods

We require information from authors about some types of materials, experimental systems and methods used in many studies. Here, indicate whether each material, system or method listed is relevant to your study. If you are not sure if a list item applies to your research, read the appropriate section before selecting a response.

Materials & experimental systems

n/a	Involved in the study	
☐	☒	Antibodies
☐	☒	Eukaryotic cell lines
☒	☐	Palaeontology
☐	☒	Animals and other organisms
☒	☐	Human research participants
☒	☐	Clinical data

Methods

n/a	Involved in the study	
☒	☐	ChIP-seq
☒	☐	Flow cytometry
☒	☐	MRI-based neuroimaging

Antibodies

Antibodies used	Rabbit polyclonal anti-laminin (Sigma L9393, lot # 067M4872V)<br>Anti-myosin (MF20, Developmental Studies Hybridoma Bank)<br>Alexa Fluor 647 goat anti-rabbit (H+L) antibody (Invitrogen A-21244)<br>Alexa Fluor 680 goat anti-mouse (H+L) antibody (Invitrogen A-21057)
Validation	Rabbit polyclonal anti-laminin: established antibody for immunofluorescent labeling of muscle fibers; see references PMID: 29581287 and PMID: 28186492 for examples.<br>Anti-myosin (MF20): established antibody that recognizes all myosin heavy chain isoforms across mammals, zebrafish, and other

species. See PMID: 12944397 or manufacturer's website for additional citations.

Eukaryotic cell lines

Policy information about cell lines

Cell line source(s)	C2C12 mouse myoblast cell line (American Type Culture Collection)
Authentication	Cell lines were not authenticated but were used within a limited passage number (approximately 10 passages maximum) once obtained from the manufacturer, and are a well-established cell line with a distinct and recognizable capacity to fuse to form multinucleated myotubes.
Mycoplasma contamination	Cell lines were not tested for mycoplasma contamination.
Commonly misidentified lines (See ICLAC register)	No commonly misidentified lines were used in this study.

Animals and other organisms

Policy information about studies involving animals; ARRIVE guidelines recommended for reporting animal research

Laboratory animals	This study used C57BL/6 wild-type male mice (Mus musculus) at a range of ages including postnatal day 10, postnatal day 21, 5-7 months, 24 months, and 30 months of age. Mice were housed at 22.2 degrees Celsius with 30-70% humidity and a 14 hr light/10 hr dark cycle. See Methods for additional details.
Wild animals	This study did not involve wild animals.
Field-collected samples	This study did not involve field-collected samples.
Ethics oversight	Animal procedures were approved by Cincinnati Children's Hospital Medical Center's Institutional Animal Care and Use Committee (IACUC2017-0053).

Note that full information on the approval of the study protocol must also be provided in the manuscript.
