# Supplementary material for: Single-nucleus RNA-seq identifies transcriptional heterogeneity in multinucleated skeletal myofibers
Source: Nat Commun. 2020 Dec 11;11:6374. doi: 10.1038/s41467-020-20063-w (PMC7733460; doi:10.1038/s41467-020-20063-w)
Supplement: Supplementary file 7 — Description of Additional Supplementary Files [file 41467_2020_20063_MOESM7_ESM.pdf]

File title: Supplementary Data 1.
Description: The top 25 marker gene lists for all individual snRNAseq datasets.

File title: Supplementary Data 2.
Description: NMJ and MTJ upregulated genes.

File title: Supplementary Data 3.
Description: Differentially expressed genes between 5 months, 24 months, and 30 months of age.

File title: Supplementary Data 4.
Description: snRNAseq quality control metrics.
